# Acceptability of Tele-mental Health Services Among Users: A Systematic Review and Meta-analysis

**DOI:** 10.1186/s12889-024-18436-7

**Published:** 2024-04-24

**Authors:** Rowan M. Abuyadek, Esraa Abdellatif Hammouda, Ehab Elrewany, Dina Hussein Elmalawany, Rasha Ashmawy, Sally Zeina, Assem Gebreal, Ramy Mohamed Ghazy

**Affiliations:** 1 Health Administration and Behavioral Sciences Department, High Institute of Public Health, Alexandria University, Alexandria, Egypt; 2 Clinical Research Department, El-Raml Pediatric Hospital, Ministry of Health and Population, Alexandria, Egypt; 3 Tropical Health Department, High Institute of Public Health, Alexandria University, Alexandria, Egypt; 4 Middle District of Health, Ministry of Health and Population, Alexandria, Egypt; 5 Department of Clinical Research, Maamora Chest Hospital, MoHP, Alexandria, Egypt; 6 Clinical Research Administration, Directorate of Health Affairs, MoHP, Alexandria, Egypt; 7 Alexandria Faculty of Medicine, Alexandria University, Alexandria, Egypt; 8 Family and Community Medicine, College of Medicine, King Khalid University, Abha, Saudi Arabia

**Keywords:** Internet based, Mental health, Tele-mental health, Health service users, Acceptability, Telehealth, Telemedicine

## Abstract

**Background:**

Mental disorders are currently a global public health concern, particularly after the coronavirus disease 2019 (COVID-19) pandemic. Mental health services gradually transitioned to teleservices, employing various methods like texting and videoconferencing. This meta-analysis aimed mainly to quantify the acceptability of tele-mental health services among both beneficiaries and providers. Secondary objectives included quantifying the usability of and satisfaction with these services.

**Methods:**

We conducted a systematic search of the following databases PubMed Central, SAGE, Google Scholar, Scopus, Web of Science, PubMed Medline, and EBSCO according to Preferred Reporting Items of the Systematic Reviews and Meta-Analysis (PRISMA) guidelines until December 2022.

**Results:**

Out of 3366 search results, 39 studies fully met the inclusion criteria. The pooled acceptability of tele-mental health services among beneficiaries was [71.0% with a 95% confidence interval (CI) of 63.0 − 78.5%, I^2^ = 98%]. Using meta-regression, four key factors contributed to this heterogeneity (R^2^ = 99.75%), namely, year of publication, type of mental disorder, participant category, and the quality of included studies. While acceptability among providers was [66.0% (95%CI, 52.0 − 78.0%), I^2^ = 95%]. The pooled usability of tele-mental health services among participants was [66.0% (95%CI, 50.0 − 80.0%), I^2^ = 83%]. Subgroup analysis revealed statistically significant results (*p* = 0.003), indicating that usability was higher among beneficiaries compared to providers.

**Conclusions:**

The study highlighted a high acceptability of tele-mental health services. These findings suggest a promising outlook for the integration and adoption of tele-mental health services and emphasize the importance of considering user perspectives and addressing provider-specific challenges to enhance overall service delivery and effectiveness.

**Supplementary Information:**

The online version contains supplementary material available at 10.1186/s12889-024-18436-7.

## Introduction

According to the World Health Organization (WHO), there is a remarkable global increase in mental disorders [1]. Approximately, 12.5% of the global population is affected by mental disorders, which can have deleterious effects on physical health, quality of life, social relationships, and occupational functioning. The prevalence of mental disorders is rising among adolescents and young adults worldwide [2]. It is worth noting that anxiety and depression are the leading causes of disability and premature mortality [3, 4]. Globally, suicide has become the fourth leading cause of death among the population aged 15–29 years, it accounts for over 700,000 deaths annually [5]. While many mental disorders can be effectively treated at a relatively low cost, there still exists a gap between the demand for care and its accessibility. This is because the effective coverage of mental health services remains very low. The WHO Special Initiative on Mental Health was launched (2019–2023), to ensure adequate access of patients with mental conditions to quality and affordable services [6].

Due to the coronavirus disease 2019 (COVID-19) pandemic, the delivery of healthcare services has remarkably changed globally [7]. To combat healthcare-associated infections, access to face-to-face or traditional consultations has been significantly limited, and the healthcare systems have resorted to remote service delivery techniques such as telehealth [8, 9]. The telehealth trend, with its potential to address many key challenges in healthcare, has been emerging worldwide. It enhanced health services accessibility, and timeliness in managing patients with physical and mental disorders. This is crucial particularly in public health crises such as epidemics and natural disasters [9, 10], in remote and rural areas, especially in countries with a large population [11]. Also, it can be used on a large scale to serve patients in isolated regions and even reach them across borders [12]. Furthermore, telehealth addresses disparities in mental health services stemming from shortages of professionals, brief visit durations, and the increasing prevalence of mental disorders [13].

Apart from diagnosis and treatment, tele-mental health also provides mental health education, psychotherapy and monitoring symptoms and adverse events. For example, changes in sleep and appetite, metabolic indicators in schizophrenic patients, and mood fluctuations in patients with bipolar disorder, which are hard to capture during short mental health professionals’ visits. This will pave the way to personalized mental health services, which in turn would enhance patients’ outcome and improve the quality of the delivered services [14].

In an Australian survey of parents, over one third of participants expressed a readiness to utilize therapist-guided and self-guided digital mental interventions, particularly to support their children [15]. A study in Mexico found that approximately half of its participants, including mental health professionals, patients, and the general population, viewed mental health services delivered via mobile phones as highly acceptable and recommended [13]. Moreover, 78.5% of Jamaican adolescents expressed interest in mobile phone-based digital interventions (mHealth) [16]. Furthermore, over 70% of African American women indicated convenience and willingness to use video calls to connect with professionals for managing anxiety and depression [17].

With growing interest in transformation of mental health services, particularly after the pandemic and among vulnerable group of beneficiaries from face to face to tele-services, it is crucial to have a comprehensive view towards user acceptability which establishes a prerequisite for usage and satisfaction, which are fundamental requirements for successful implementation of tele-mental health services. Through this meta-analysis, we aimed to cast light on the acceptance of implementing tele-mental health services (online, web-based interventions) among users. Additionally, it evaluated the usability and satisfaction of these services. The study findings would give the decision makers and other stakeholders a big hand in planning and executing more strategies to assure both availability and accessibility to tele-mental health services.

## Methods

We performed this systematic review in strict compliance with the preferred reporting items of the systematic review and metanalysis (PRISMA) checklist. All steps were conducted in concordance with the Cochrane Handbook of systematic review and metanalysis [18].

### Operational case definitions

- *Acceptance of healthcare interventions*, which is “a multi-faceted construct that reflects the extent to which people delivering or receiving a healthcare intervention consider it to be appropriate, based on anticipated or experienced cognitive and emotional responses to the intervention” [19].
- *Usability* is “the effectiveness, efficiency, and satisfaction with which users achieve goals in a specific environment” []. In healthcare, the “particular environment” mentioned in the definition can be: care settings, medical devices, software, workflows, and related processes [20]. The Healthcare Information and Management Systems Society [21] stated the main principals of usability of medical software are: simplicity, naturalness, consistency, minimizing cognitive load, efficient interactions, forgiveness and feedback, effective use of language, effective information presentation, and preservation of context [21].
- *Satisfaction* is one of the key metrics used to assess service quality and usability [22]. Patient satisfaction embodies the patient's perceived need, expectations from the health system, and experience of health care. This multidimensional concept includes both medical and non-medical aspects of health care [23]. The acceptability of tele-mental health services among users denotes their willingness and openness to employ these services for their mental health care and treatment. In contrast, usability evaluates the ease of use and interaction design of the telehealth systems. Both acceptability and usability impact user satisfaction with their experience and outcomes when utilizing tele-mental health services.

### The study outcomes

- *Primary outcome*: Quantifying acceptance of tele-mental health services among its users either patients, providers, payors, or healthcare policy makers.
- *Secondary outcomes* of the study include:
  - Assessment of the usability of tele-mental health services.
  - Evaluation of satisfaction levels with tele-mental health services.

### Inclusion and exclusion criteria

The inclusion criteria for articles in the study encompassed a broad spectrum: all articles had to address the acceptance of tele-mental health services, characterized by mental health services provided when the patient and provider are not physically co-located, facilitated through web-based or mobile-based tools. The scope of mental disorders was inclusive, and any target population, comprising users of tele-mental health services including patients, providers, payors, and policy makers was considered eligible. Additionally, studies of any design, whether observational or interventional, were included, provided that they measured the acceptance of the service. There was no specified time limit for the search period.

The exclusion criteria for articles in the study were defined as follows: Short Message Service (SMS) based services were excluded as it isn't aligning with the definition of tele-mental health services. Articles written in languages other than English were also excluded. Additionally, case report, case series, editorial, letter to editor, conference abstract papers, paper can’t be accessed, and data can’t be extracted were excluded.

### Search strategy

We searched EMBASE, Scopus, EBSCO, MEDLINE central/PubMed, SAGE, Web of Science, and Google Scholar databases for articles without timeframe or geographical restrictions till December 20, 2022. (Table S1).

### Data organization, extraction, and quality assessment

- All records were exported to an Endnote library to detect and remove duplicates using the “remove duplicate function”. All references that had the same title and author and were published in the same year or the same journal were removed. References remaining after this step were exported to a Microsoft Excel file with essential information for screening.
- The title and abstract screening were done by two independent reviewers to select papers based on the inclusion criteria.
- During the full-text screening phase, all selected articles were downloaded, and the full text was reviewed by two independent reviewers. If any disagreement was noticed, the senior author (RMG) was asked to make his decision.
- Interrater agreement for both title and abstract screening, and full text screening were calculated and displayed as Cohen’s Kappa.
- We applied backward and forward search methods to do manual searching. Backward search enclosed searching the reference lists of all included articles. Forward search involved citation tracking in which the reviewers tracked all the articles that cite each one of the included articles, and all ‘related to’ or ‘similar’ articles. All excluded records were given exclusion reasons.
- The data extraction was then thoroughly conducted by five reviewers (RMA, EE, EAH, DHE & AG) and checked by RA. Data were synthesized using Microsoft Office 2016 Excel spread sheet as well as narrative format to extract (First author last name, year, study setting, type of study either face-to-face or online survey, sample size and demographics, type of population either patients, healthcare providers, payors, or policy makers). As a primary objective, mean or proportion of acceptance of tele-mental health services, tool used for assessment of acceptance and the method of its delivery, either online or face to face, were extracted. Also, type on mental health disorder targeted by the tele- services including mood and/or anxiety disorders, psychotic, personality, and substance related and addictive disorders, and severe mental disorders. Additionally, the type of tele-mental health service delivery either mobile application, mobile game, video conferencing, video consultation, social media plateform or others were also extracted. Type of intervention either diagnostic or therapeutic or both was also retrieved.
- As additional objectives, mean and/or proportion of both usability and satisfaction and their data collection tools. Moreover, data regarding the type of remote intervention either diagnostic or therapeutic and the tool used either, video-based calls, mobile application or others were extracted whenever possible.
- The quality of each study was examined based on a set of methodological criteria for such studies previously suggested by Newcastle-Ottawa Scale by two different reviewers and Interrater agreement was also calculated and displayed [24].

### Statistical analysis

- **Publication bias**: was assessed by visual inspection of the funnel plot. Publication bias was explored by Begg’s test, a *p-value <* 0.05 was defined, a priori, to indicate the possible presence of publication bias.
- **Quantitative data synthesis**: Data extracted from the selected studies for each category were pooled in metanalyses by use of R software version 4.2 (meta and metafore packages). Data were reported as proportion, hence they were pooled using pooled prevalence, with the perspective of a 95% confidence interval (CI) in the meta-analysis model. In the case of zero frequency, the correction value of 0.1 was used. Random effect models were used if the test of heterogeneity for a group of study results was significant (defined conservatively as *p* < 0.20).
- Forest plots were presented to visualize the degree of variation of effect size between studies.
- **Assessment of heterogeneity**:
  - Visual inspection of the forest plot.
  - Cochrane I-Square test (I^2^), following Cochrane Handbook for Systematic Reviews of Interventions (I^2^), and it was interpreted as follows: “0–40%: might not be important; 30–60%: may represent moderate heterogeneity; 50–90%: may represent substantial heterogeneity; 75–100%: considerable heterogeneity. The importance of the observed value depends on the magnitude and direction of effects, and strength of evidence for heterogeneity.
  - Meta-regression analysis was used to assess how the inclusion of predictors namely, year of publication, mental disorder type, participant category (patients, caregivers, general population, refugees) influenced the effect size of the studies, thereby explaining the significant heterogeneity observed.
  - Leave one out sensitivity analysis. We conducted a leave-one-out sensitivity analysis using the metafor R tool. This involved recalculating the meta-analysis results K times while excluding one study each time. To identify influential studies, we used the influence () function, which includes a series of leave-one-out diagnostic tests. We categorized what we considered to be influential and sorted the studies in the plot using I^2^.
  - Remove outlier function: If a study’s confidence interval does not align with that of the pooled effect, it’s identified as an outlier. These outliers significantly impact the overall effect and deviate notably from the aggregate result. Studies with high sampling error often diverge substantially from the pooled outcome. However, due to their wide confidence intervals, there’s a higher likelihood of overlap with one of the pooled effects. This fundamental technique for outlier detection is implemented using the “find outliers” function. It identifies and removes outlier studies within a meta-analysis, subsequently recalculating the result.
  - Subgroup analysis: we categorized the usability of tele mental health services according to the type of user, either beneficiary or provider.

### Results

**Fig. 1:**
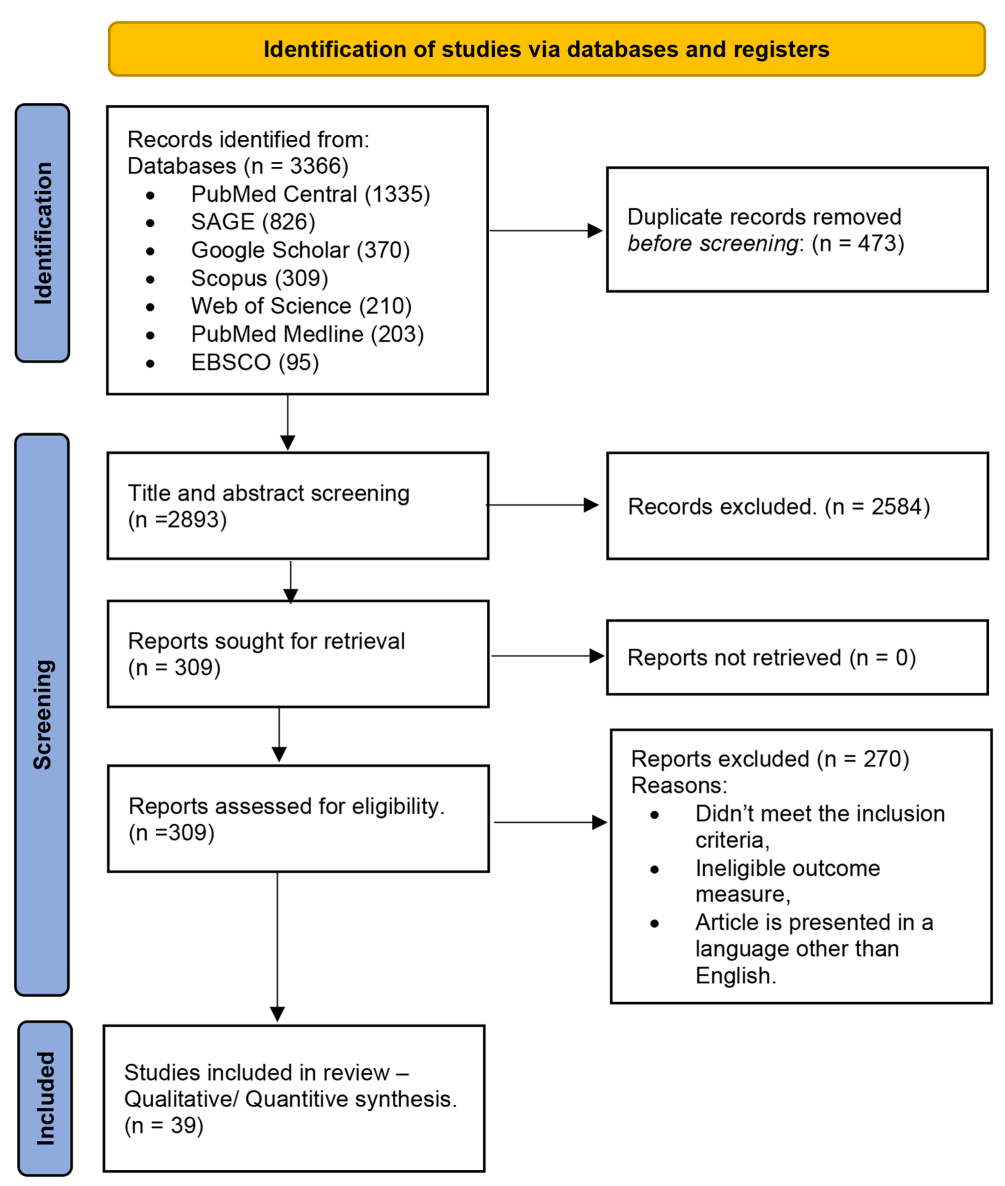
PRISMA flow diagram for new studies which included searches of databases

The PRISMA flow diagram summarized the study selection process. The included database’s search yielded a total of 3366 articles, before screening, 473 articles were removed for being duplicates. The screening phase involved 2893 articles proceeded to title and abstract screening, out of them, 2584 were removed as irrelevant and not coincident with inclusion criteria. Overall, 309 articles were eligible for full text screening and all of them were retrieved. Two hundreds and seventy articles were removed as they didn’t meet the inclusion criteria, ineligible outcome measure, article is presented in a language other than English. No new studies were encountered through the manual backward and forward search Fig. 1. The agreement between reviewers in title and abstract screening, full text screening, and quality assessment were substantial (k = 0.80, 0.88, 0.85), respectively.

A total of 39 studies published between 2004 and 2022 were included in the review, covering wide geographical locations and different types of mental disorders. Nine studies were conducted in the United States of America (USA) [17, 25–32], seven studies were conducted in Germany [33–40], three studies were conducted in the United Kingdom (UK) [41–43] and Australia [15, 16, 44], two studies were conducted in Netherlands [45, 46], France [47, 48] and China [49, 50], one study in Canada [51], Austria [33], Sweden [52], New Zealand [16], Portugal [53], and Estonia [54]. Studies from Africa included Egypt [40], Nigeria [55], Mozambique [56], and Kenya [57]. Additional one study was conducted in India [58] and in Mexico [13]. The studies’ populations were further classified as either healthcare beneficiaries, providers, or both. Studies enclosed different types of healthcare beneficiaries [13, 15, 17, 25, 27–30, 32–36, 38–41, 43–47, 50, 53–55, 57, 59] namely, general population, students, refugees, patients, pregnant women, parents of children who completed cancer treatment and other caregivers. Providers were represented either as healthcare workers (HCWs) or caregivers [16, 31, 37, 38, 51, 56, 58]. Some studies included both populations of providers and beneficiaries [26, 42, 48]. Studies covered many types of mental disorders, such as anxiety [13, 15, 17, 32, 35, 37, 44–46], mood changes [13, 15, 17, 25, 27, 30, 37, 39, 44–46, 53, 55, 59], psychotic disorders [27, 28, 33, 43, 50], neurocognitive disorders [26, 48], personality disorders [31], substance-related and addiction [29, 56, 57], severe mental illness [29, 58], and other non-specified mental disorders [16, 34, 36, 38, 40–42, 47, 49, 51, 52, 54]. Acceptability of tele-mental health services was studied for either educational [55], therapeutic [13, 15–17, 25, 28, 29, 31, 33–39, 42, 44–49, 52, 54, 56, 58], or diagnostic [26, 30, 41, 43, 59] services or both [27, 32, 40, 50, 51, 53, 57], Table 1.

**Table 1:** All published studies that reported acceptability of tele-mental health services

Study	Year	Participants	Age category	Mental disorder	Acceptance rate or Mean ± SD	Tool used	Method of application	Usability rate	Type of intervention	Quality
	Country	Classified as;		Classified as;		Type of delivery				
	Design							Satisfaction		
Dietvorst [45]	2022, Netherlands, Cross-sectional	Students	Adolescences	Anxiety and depression	72.60%	Questionnaire	Mobile application		Therapeutic	Satisfactory
Dietvorst [45]					75.60%					
Dietvorst [45]		Beneficiary		Mood Disorders; anxiety disorders	7.1 ± 1.5 (from total score 10)	Online				
Ericze´n [25]	2012	Pregnant	Adults	Maternal depression	97.00%	Questionnaire	E-mental health interventions		Therapeutic	Satisfactory
	USA	Beneficiary		Mood disorders		Mixed				
	Cross-sectional									
Mccall [17]	2019	Patients	Adults	Anxiety and depression	70.00%	Questionnaire	Mobile phone		Therapeutic	Satisfactory
	USA	Beneficiary		Mood disorders; anxiety disorders		Online				
	Cross-sectional									
Eichenberg [33]	2016	Patients	Adults	Psychological condition needs further investigation	61.90%	Questionnaire	Serious game application		Therapeutic	Good
	Austria and Germany	Beneficiary		Psychotic disorders		Online				
	Cross-sectional									
Woodford [52]	2018	Parents of children who had completed cancer treatment.	Adults	Mental health disorders	74.10%	Questionnaire	Psychological interventions by internet		Therapeutic	Good
	Sweden	Beneficiary		Non specified		Online				
	Cross-sectional									
Lincke [34]	2022	General population	All	Mental health disorders	20.00%	Questionnaire	E-mental health interventions		Therapeutic	Good
	Germany	Beneficiary		Non specified		Online				
	Cross-sectional									
Proudfoot [44]	2010	General population	Adults	Anxiety and depression	76.00%	Questionnaire	Mobile phone		Therapeutic	Satisfactory
	Australia	Beneficiary		Mood disorders; anxiety disorders		Online				
	Cross-sectional									
Damerau [35]	2021	Patients	Adults	Generalized anxiety disorder	3.02 ± 1.14	Questionnaire	E-mental health interventions		Therapeutic	Good
	Germany	Beneficiary		Anxiety disorders		Online				
	Cross-sectional									
Greenwood [59]	2004	Patients	Adults	Overwhelmingly mood disorders	80.00%	Questionnaire	Telepsychiatry	**(12) 60%**	Diagnostic	Unsatisfactory
	--	Beneficiary		Mood disorders		Mixed				
	Cross-sectional							(19) 95%		
Bruen [41]	2020	Patients	Adults	Mental health disorders	86.00%	Data from app.	Swim App	**67 (84%)**	Diagnostic	Good
	UK	Beneficiary		Non- specified						
	Cross-sectional									
Hagen [36]	2020	Patients	Adults	Perceived stress, cognitive attitudes	2.76 ± 1.16	Questionnaire	Stress prevention programs		Therapeutic	Good
	Germany	Beneficiary		Non- specified		Mixed				
	Cross-sectional									
Kenter [46]	2013	Patients	Adults	Anxiety and depression	53.00%	Questionnaire	Problem solving treatment		Therapeutic	Satisfactory
	Netherlands	Beneficiary		Mood disorders; anxiety disorders		Online				
	Observation									
Deb [58]	2018	Caregivers	Adults	Severe mental illness	62.50%	Questionnaire	Mobile application	**16/34 (47.1%)**	Therapeutic	Satisfactory
	India	Provider				Face to Face				
	Cross-sectional									
Cloutier [51]	2008	HCWs	Adults	Mental health disorders	92.00%	Questionnaire	Video conferencing		Diagnostic and Therapeutic	Satisfactory
	Canda	Provider		Non- specified		Online				
	Cross-sectional									
Kiburi [57]	2022	Patients	Adults	Opioid use disorders	95.00%	Questionnaire	Digital interventions		Diagnostic and Therapeutic	Satisfactory
	Kenya	Beneficiary		Substance-related and addictive disorders		Face to Face				
	Cross-sectional									
Kola [55]	2021	Pregnant	Adolescences	Maternal depression	96.20%	Questionnaire	Mobile application/ text messages		Educational	Satisfactory
	Nigeria	Beneficiary		Mood disorders		Face to Face				
	Cross-sectional									
A. O’Grady [56]	2021	HCWs	Adults	Substance use (alcohol)	4.33(± 0.45)	Questionnaire	Smartphone		Therapeutic	Satisfactory
	Mozambique	Provider		Substance-related and addictive disorders		Online				
	Cross-sectional									
Linardon [15]	2021	Caregivers	Adults	Anxiety and depression	30.00%	Questionnaire	Mobile phone		Therapeutic	Good
	Australia	Beneficiary		Mood disorders; anxiety disorders		Online				
	Cross-sectional									
Sander [37]	2021	HCWs	Adults	Anxiety and depression	36.00%	Questionnaire	Online platform		Therapeutic	Good
	Germany	Provider		Mood disorders; anxiety disorders		Face to Face				
	Cross-sectional									
Tan [49]	2020	Patients– HCWs - General population	All	Mental health disorders	50.00%	Questionnaire	Social media, Smartphone apps, Websites		Therapeutic	Satisfactory
	China	Beneficiary & Provider		Non-specified		Online				
	Cross-sectional									
Hendrikof [38]	2018	Patients	Adults	Mental health disorders	49.00%	Questionnaire	Video consultation		Therapeutic	Satisfactory
	Germany	Beneficiary		Non-specified		Mixed				
	Cross-sectional									
Hendrikof [38]	2018	HCWs	Adults	Mental health disorders	56.20%	Questionnaire	Video consultation		Therapeutic	
	Germany	Provider		Non-specified		Mixed				
	Cross-sectional									
Landes [31]	2021	HCWs	Adults	Personality disorder	73.00%	Questionnaire	Non-specified		Therapeutic	Unsatisfactory
	USA	Provider				Online				
	Cross-sectional									
Farrer [16]	2022	HCWs (80.1% psychologist- 9.7% social workers- 5.3% counsellor)	Adults	Mental health disorders	73.00%	Questionnaire	Non-specified	57.7%	Therapeutic	Satisfactory
	Australia and New Zealand	Provider		Non-specified		Online				
	Cross-sectional									
Harrell [26]	2013	Patients and caregivers	Geriatrics	Cognitive & psychological problems	100.00%	Questionnaire	Videoconferencing		Diagnostic	Satisfactory
	USA	Beneficiary & Provider		Neurocognitive disorders		Face to Face				
	Cross-sectional									
Cormi [47]	2021	HCWs	Adults	Mental health disorders	38.00%	Questionnaire	Non-specified		Therapeutic	Good
	France	Beneficiary		Non-Specified		Online				
	Cross-sectional									
Li [27]	2022	Patients	Adults	Anxiety and psychotic disorders	87.00%	Questionnaire	Videoconferencing		Diagnostic and Therapeutic	Satisfactory
	USA	Beneficiary		Mood disorders; psychotic disorders		Telephone- based				
	Cross-sectional									
Lynch [28]	2020	Patients	Adults	Complex psychosis	89.00%	Questionnaire	Non-specified		Therapeutic	Satisfactory
	USA	Beneficiary		Psychotic disorders		Mixed				
	Cross-sectional									
Mehrabian [48]	2014	Patients and caregivers.	Geriatrics	Cognitive impairment and Alzheimer’s disease	65.00%	Questionnaire	Non-specified		Therapeutic	Satisfactory
	France	Beneficiary & Provider		Neurocognitive disorders		Face to Face				
	Cross-sectional									
Benjet [13]	2020	Students	Adolescences	Anxiety and depression	48.00%	Questionnaire	Mobile applications		Therapeutic	Good
	Mexico	Beneficiary		Mood disorders; anxiety disorders		Web Based				
	Cross-sectional									
Painter [42]	2021	Patients and HCWs	Adults	Mental health disorders	84.00%	Questionnaire	Video consultation		Therapeutic	Unsatisfactory
	UK	Beneficiary & Provider		Non-specified		Telephone- based				
	Cross-sectional									
Gowarty [29]	2021	Patients	Adults	Severe mental illness, smoking cessation	58.80%	Questionnaire	Mobile applications	Moderate to high satisfaction	Therapeutic	Unsatisfactory
	USA	Beneficiary		Severe mental illness; Substance-related and addictive disorders		Face to Face				
	Mixed Methods									
Tark [54]	2019	Patients	Paediatrics	Mental health disorders	78.00%	Questionnaire	Mobile game		Therapeutic	Unsatisfactory
	Estonia	Beneficiary		Non-Specified		Face to Face				
	Cross-sectional									
Guarino [32]	2021	Patients and healthy population	Adults	Anxiety and depression	5.90 (± 0.4).	Questionnaire	Web-based program		Diagnostic and Therapeutic	Satisfactory
	USA	Beneficiary		Mood disorders; anxiety disorders		Online				
	Cross-sectional									
Mayer [39]	2022	Patients	Adults	Depression	0.25 ± (1.04)	Mixed	Mobile applications		Therapeutic	Good
	Germany	Beneficiary		Mood disorders		Face to face				
	Cross-sectional									
Burchert [40]	2019	Refugees	Adults	Mental health disorders		Questionnaire	Web-based program		Diagnostic and Therapeutic	Satisfactory
	Germany, Sweden And Egypt	Beneficiary		Non specified		Face to Face				
	Cross-sectional									
Cella [43]	2017	Patients and healthy population	Adults	Schizophrenia	80.000%	Questionnaire	Web-based program		Diagnostic	Satisfactory
	UK	Beneficiary		Psychotic disorder		Face to Face				
	Cross-sectional									
Williams [30]	2014	Students	Adolescences	Depression	93.80%	Questionnaire	Web-based program		Diagnostic	Unsatisfactory
	USA	Beneficiary		Mood disorders		Online				
	Cross-sectional									
Xiao [50]	2020	Patients	Adults	Schizophrenia	43.00%	Questionnaire	Web-based program		Diagnostic and Therapeutic	Satisfactory
	China	Beneficiary		Psychotic disorder		Face to Face				
	Cross-sectional									
Fonseca [53]	2016	Pregnant women	Adults	Depression	Mean 2.73 (0.63)	Questionnaire	Web-based program		Diagnostic and Therapeutic	Satisfactory
	Portugal	Beneficiary		Mood disorders		Online				
	Cross-sectional									

Abbreviation list: App; Application, e; Electronic, HCWs; Healthcareworkers, UK: United Kingdom, USA: United States of America

Usability values are shown in **bold**

### Acceptance of tele-mental health services

#### Acceptance of tele-mental health services among beneficiaries

**Fig. 2:**
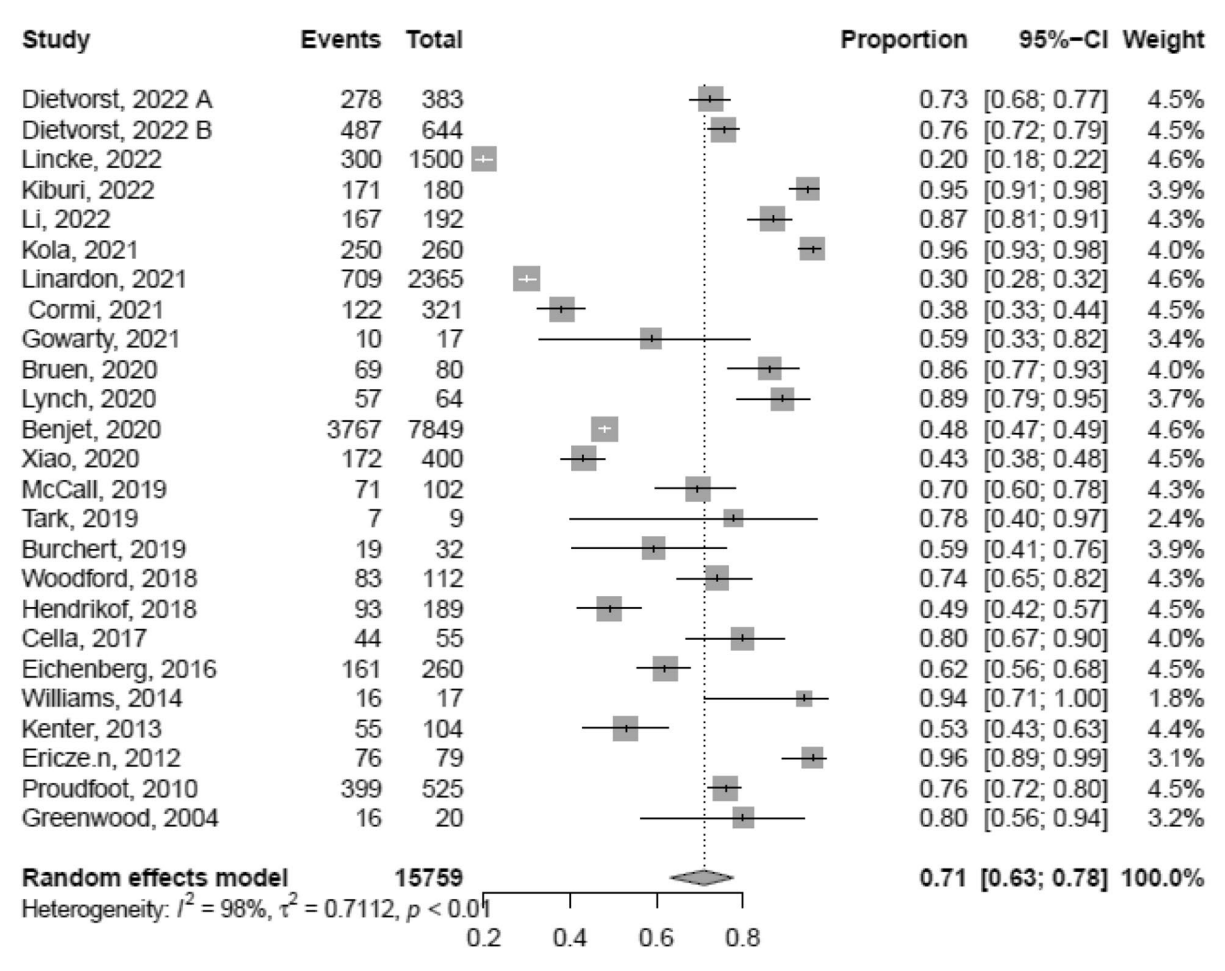
Forest plot showing acceptance of tele-mental health services among beneficiaries

Based on the findings of 24 studies conducted from 2004 to 2022 [10, 13, 15, 28–30, 33, 34, 38, 40, 41, 43, 45–47, 50, 52, 54, 55, 57, 59–62], the pooled acceptance of tele-mental health services among beneficiaries was 71.0% with a 95% confidence interval (CI) of 63.0 − 78.5%, I^2^ = 98%. Acceptance of tele-mental health services ranged widely across the studies, from as low as 20% (95%CI, 18.0 − 22.0%) to as high as 96% (95%CI, 89.0 − 99.0%). Thus, a meta-regression was performed to explain this heterogeneity including year of publication, mental disorder type, participant category (patients, caregivers, general population, refugee) and the quality of included studies contributes (R^2^ = 99.75%), Fig. 2.

**Fig. 3:**
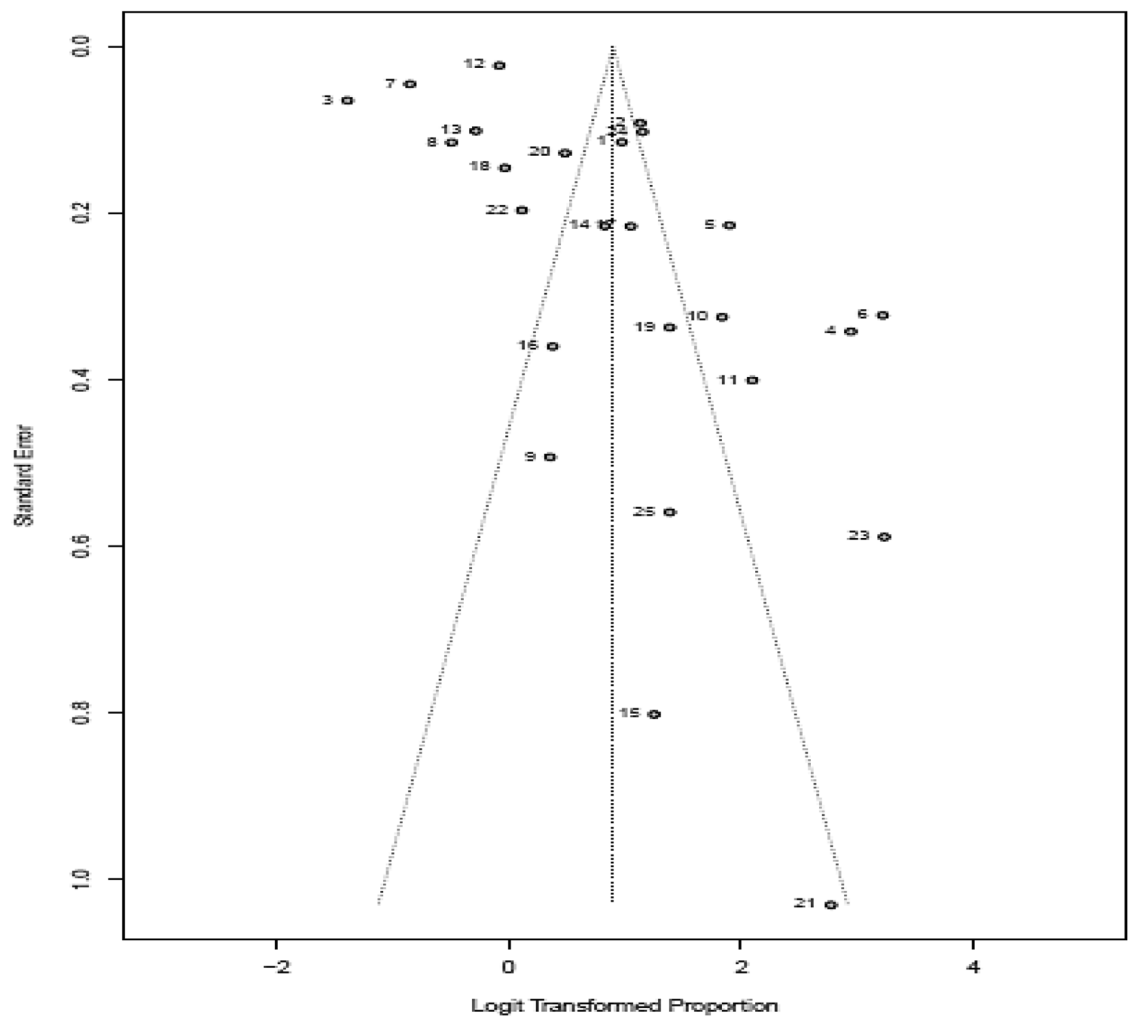
Funnel plot showing acceptance of tele-mental health services among beneficiaries

Visual inspection of funnel plot showed that studies with higher effect size are concentrated at the top of the pot. The Egger’s test result shows a value of 3.4 and a p-value of 0.002 indicating the presence of funnel plot asymmetry. This asymmetry may have implications for the interpretation of the studies and their associated evidence, indicating the possibility of publication bias or other sources of systematic bias, Fig. 3.

#### Acceptance of tele-mental health services among providers

**Fig. 4:**
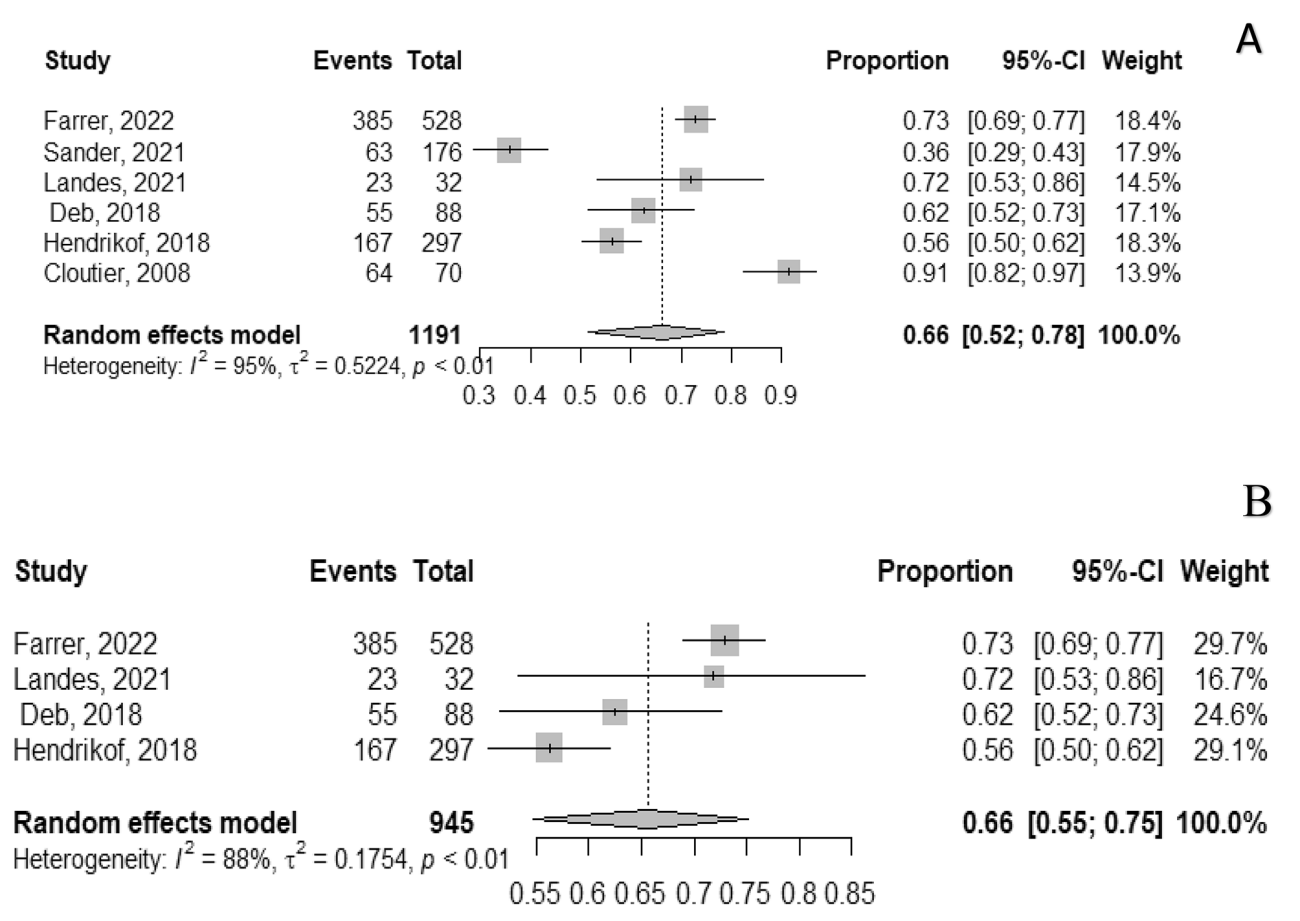
Forest plot showing acceptance of tele-mental health services among providers, after removal of two outlier studies (Sander, 2021, Cloutier 2008)

Based on the findings of the 6 studies conducted from 2008 to 2022 [16, 31, 37, 38, 51, 58], the pooled acceptance of tele- mental health services among providers was 66.0%, (95%CI, 52.0 − 78.0%), I^2^ = 95%. The wide range of acceptance rates across the studies is evident, with the lowest acceptance reported at 36.0% (95%CI, 29.0- 43.0%) [37], and the highest acceptance at 91.0% (95%CI, 82.0 − 97.0%) [51], Fig. 4-A. After removal of outliers’ studies, Sander 2021, and Cloutier, 2008 the pooled acceptance was 66.0% (95%CI, 55.0 − 75.0%), and heterogeneity dropped to I^2^ = 88.0%, Fig. 4-B.

**Fig. 5:**
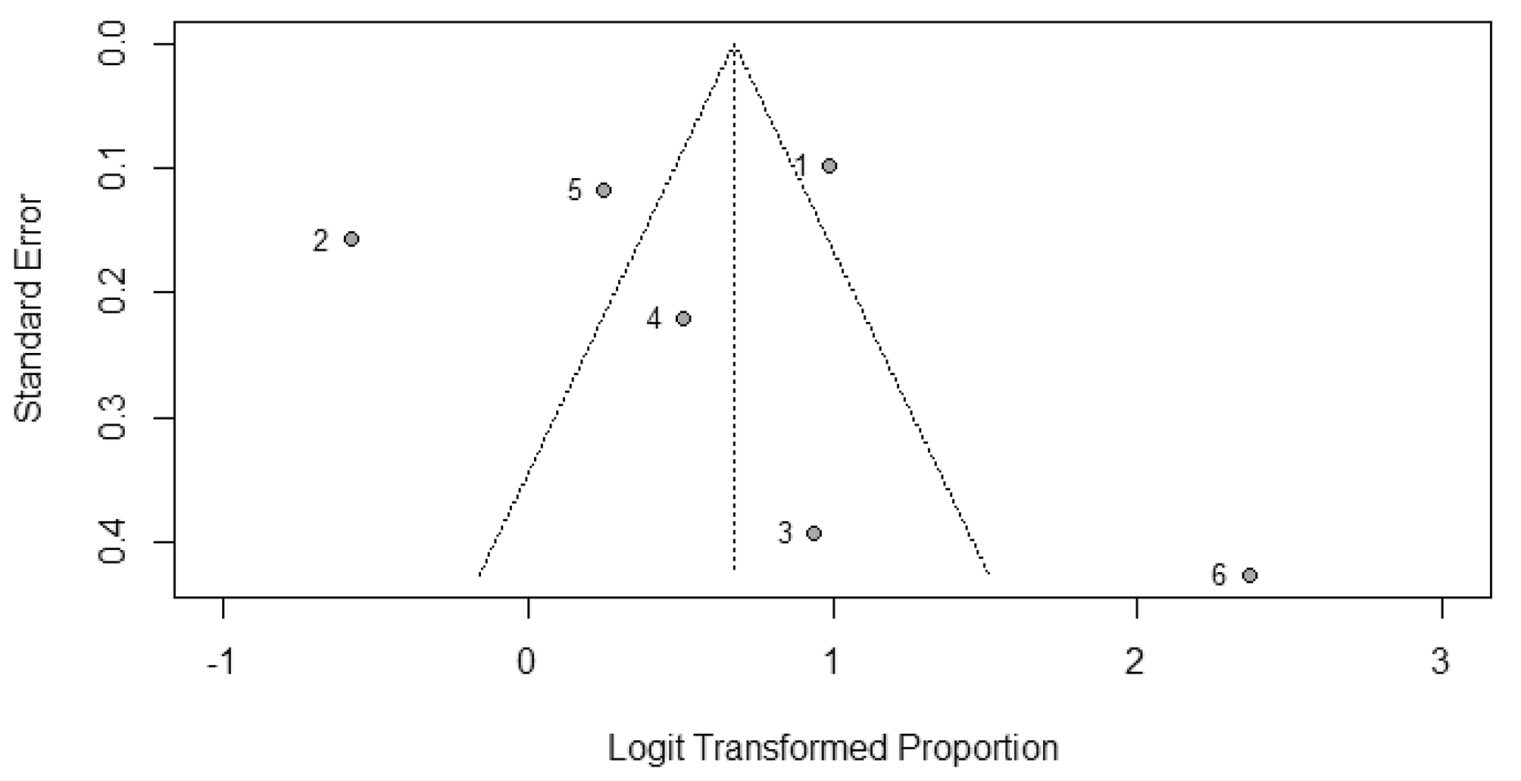
Funnel plot showing acceptance of tele-mental health services among providers

Visual inspection of the funnel plot revealed absence of publication bias. The Egger’s test result showed a (*p*-value = 0.838) that there is no evidence of funnel plot asymmetry, Fig. 5.

#### Acceptance of tele-mental health services among both (providers & beneficiaries)

**Fig. 6:**
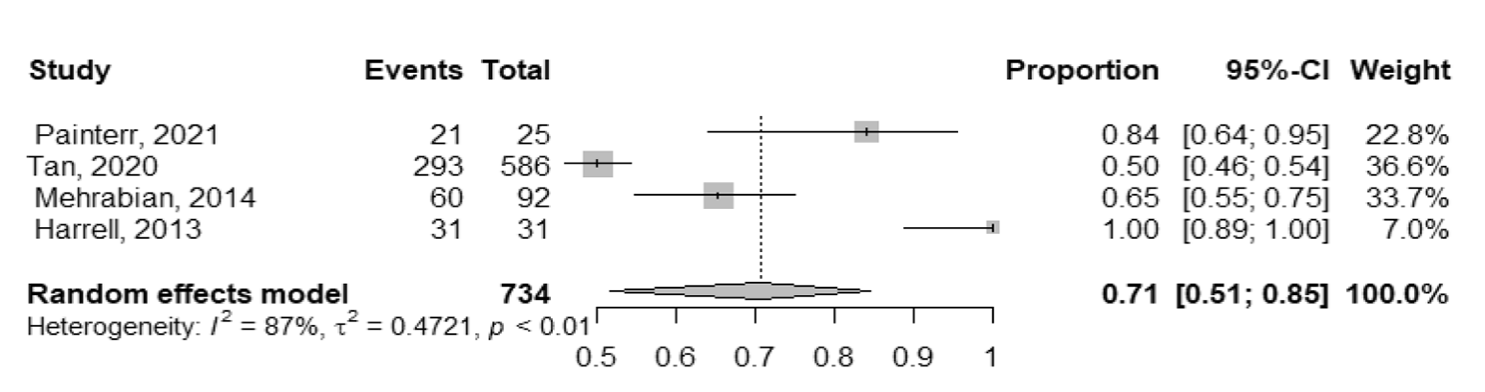
Forest plot showing acceptance of tele-mental health services among both providers and beneficiaries

Concerning the acceptance of tele- mental health services in studies involving both providers and beneficiaries, there were a total of four studies conducted in 2013–2021 [26, 42, 48, 49], encompassing 734 participants. The pooled acceptance rate was 71.0% (95%CI, 51.0 − 85.0%), I^2^ = 87%, Fig. 6. However, after conducting a leave-one-out sensitivity analysis and excluding the study of Tan 2021 [49], the heterogeneity decreased to 77.0% and pooled acceptance raised to 83.0% (95%CI, 55.0 − 95.0%).

### Usability of tele-mental health services

**Fig. 7:**
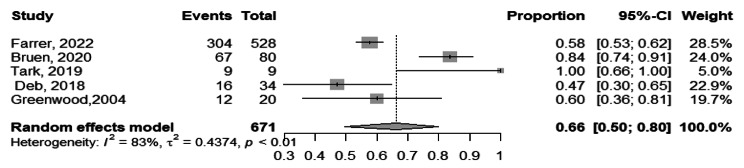
Forest plot showing usability of tele-mental health services among participants

Based on the findings of the 5 studies [16, 41, 54, 58, 59], the pooled usability of tele-mental health services among the participants was 66.0% ranging from 47.0%, 100.0% with (95%CI, 50.0 − 80.0%), I^2^ = 83.0%, Fig. 7.

**Fig. 8:**
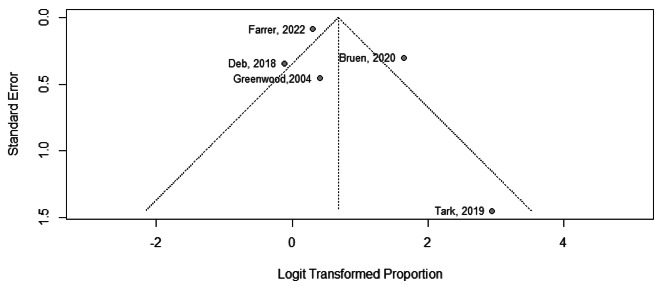
Funnel plot showing usability of tele-mental health services among participants

The visual inspection of the funnel plot, combined with Egger’s test result (*p*-value of 0.401), suggest that there is no evidence of publication bias, Fig. 8.

**Fig. 9:**
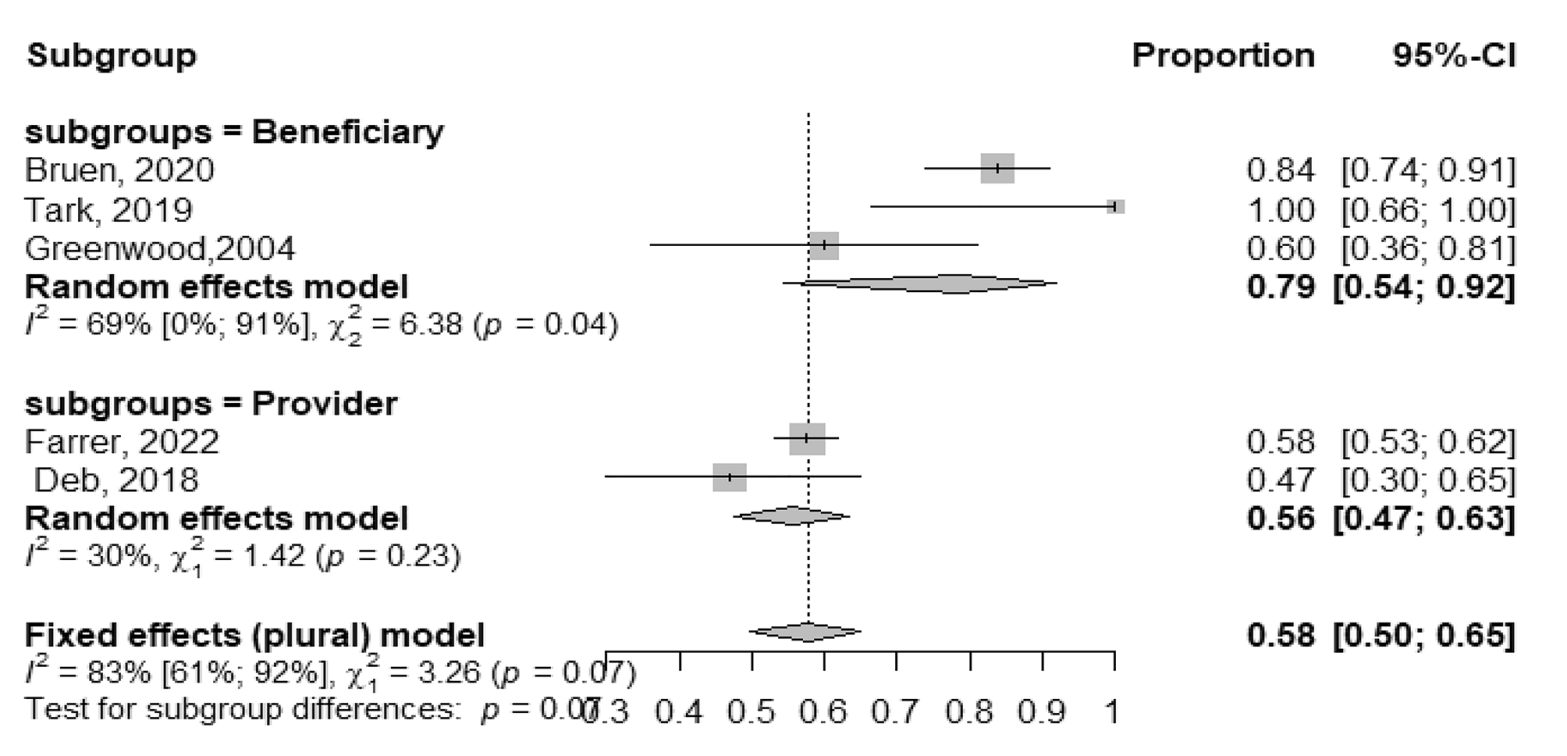
Forest plot showing usability of tele-mental health services among participants

The subgroup analysis of usability of tele-mental health services among providers and beneficiaries yielded statistically significant results (*p* = 0.003), indicating that the subgrouping factor had a significant impact on the usability outcomes. Among beneficiaries [41, 54, 59], the pooled usability of telemedicine was found to be 79.0% (95%CI, 54.0 − 63.0%), I^2^ = 69.0%. On the other hand, among providers [16, 58], the pooled usability of telemedicine was 56.0% (95%CI, 47.0 − 53.0%), I^2^ = 30%, Fig. 9.

## Discussion

Tele-mental health has proven to be effective in managing common mental health disorders. Effectiveness of videoconferencing psychotherapy for the treatment of depression and anxiety disorder revealed a promising outcome in reducing depressive and improving anxiety symptoms [63, 64]. Also, mobile applications were effectively capable of well-assisting patients to reduce disease-related symptoms of mania, depression, and psychosis [65]. Hence, this review aimed mainly to quantify the acceptability of tele-mental health services among users namely, beneficiaries and providers, to enlighten health care decision at payor and policy makers levels about expanding the provision of these services on a broader scale. Secondary objectives included quantifying the usability of and satisfaction with these services. It assessed the acceptance of tele-mental health services for various mental disorders, including mood, anxiety, psychotic, personality, substance-related, and severe mental illness. The services were delivered through mobile applications, gaming, teleconferencing, video calls, and web-based programs. The multiple interfaces played by the tool or application make it more flexible as preventive, curative and it also provides a closer monitoring of the patient. This can be enhanced soon with inclusion of artificial intelligence in preventing risky behaviors or exacerbations of the mental health conditions. This review included 39 studies, over half of them were conducted after COVID-19 pandemic. Eleven studies revealed good quality, 22 studies were satisfactory, and only six studies were unsatisfactory. As pooled measure revealed high acceptability among the users, this would potentially encourage the delivery of interventional programs to deliver this type of services especially with the encountered proper usability and satisfaction.

Similar reviews were conducted to assess the acceptability of several forms of mental health services. Shek et al. [66], published a systematic review to assess acceptability and feasibility of technology-based interventions to support mental health after stroke revealed high acceptability, satisfaction, and adherence among these patients’ group. Additionally, Grist et al. [67], conducted a systematic review of studies involving children and adolescents less than 18 years to systematically appraise efficacy and acceptability of mobile apps for mental health They found that feasibility outcomes suggest high acceptability and moderate usage of this services. On the other hand, a scoping review was conducted by Apolinário-Hagen [68] to identify and evaluate the empirical evidence of public acceptability and attitudes towards e-mental health therapies, clarified that intentions to use this services was less than face-to-face services. An umbrella review was conducted to critically appraise the published reviews about Computerized Cognitive Behavioral Therapy revealed that this service must be individually tailored before being introduced to the users, to enhance its usability and adherence [69]. These reviews present a nuanced view of the acceptability of various mental health interventions, illustrating the opportunities and challenges in meeting the diverse needs of populations. Going forward, incorporating these insights into policy and practice has the potential to enhance the delivery and acceptance of mental health services. This integration can ultimately lead to improved outcomes for individuals in search of support and care.

Tele- health services were also used in other types of care. It yielded similar outcomes as face-to-face services in the management of heart failure, it could improve the control of blood glucose levels in patients with diabetes [70], and it improved the symptom management among rural palliative care population [71]. It is also acceptable and feasible in diagnosis and treatment of Human Immune Deficiency Viral infection among adults [72]. Compared to face-to face rehabilitation services, tele-rehabilitation yielded statistically significant improvements in quality of life, chronic respiratory disease and selfcare among community-dwelling patients with chronic diseases [73]. A survey for assessment of feasibility, acceptability, and usability of telehealth visits revealed that, vast majority of respondents (98%) were comfortable with this services [74].

Delivery of this tele-health services would undoubtedly improve accessibility especially with high acceptability among different categories of users, as this review clarified, among different age group or populations at risk e.g., care givers, pregnant women. Although clinical effectiveness was not studied in this review and it was claimed to be not improving over time [75], the growing penetration of this service and its intense use among adolescents and high acceptability among this age group is a good indicator of the potential growth of this service utilization over years.

### Strengths & limitations

This review is one of the few studies that quantified perspectives of users regarding tele-mental health services acceptability, usability, and satisfaction. However, this review was limited to quantitative observational study designs with different tools which lead to high heterogeneity. Qualitative study designs are needed to provide in-depth insights regarding provision of these types of services particularly concerns related to safety and privacy. Also, this review didn`t investigate facilitators and barriers to acceptability and usability of tele-mental health services. A supporting review is needed to quantify the clinical effectiveness of this type of service before its full implementation in a healthcare system. In addition, articles written in language other than English were not included. Finally, we didn`t include grey literature and unpublished data, however, we performed a strict search of seven databases to get all the published data.

## Conclusions

This systematic review and metanalysis addressed the global landscape of tele-mental health services, particularly in the post-COVID-19 period. Our findings suggest a promising perspective of the integration and adoption of tele-mental health services and underscore a distinction between beneficiaries and providers. Policy recommendations must encompass user directed interventions and training to facilitate the seamless integration of tele-mental health into healthcare systems. Further research should address concerns and barriers faced by providers in utilizing tele-mental health services.

### Electronic supplementary material

Below is the link to the electronic supplementary material.

Supplementary Material 1

## Data Availability

All data generated or analyzed during this study are included in this published article and its supplementary information files.

## Declarations

### Registration

Prospero registration was limited during the 2020 pandemic, to prioritize COVID-19 registrations.

### Use of artificial intelligence (AI)

AI technologies were cautiously employed only to enhance the readability of this article.
