# Supplementary material for: Acceptability of Tele-mental Health Services Among Users: A Systematic Review and Meta-analysis
Source: BMC Public Health. 2024 Apr 24;24:1143. doi: 10.1186/s12889-024-18436-7 (PMC11040906; doi:10.1186/s12889-024-18436-7)
Supplement: Supplementary file 1 — Supplementary Material 1 [file 12889_2024_18436_MOESM1_ESM.docx]

**Supplementary:**

Table (S1) Search Strategy among databases

| **Data base** | **Key words** | **Frequency** |
| --- | --- | --- |
| PubMed Central | ( ( ( ( "Mental Disorder" ) OR ( "Psychiatric Illness" ) OR ( "Psychiatric Illnesses" ) OR ( "Psychiatric Diseases" ) OR ( "Psychiatric Disease" ) OR ( "Mental Illness" ) OR ( "Illness, Mental" ) OR ( "Mental Illnesses" ) OR ( "Psychiatric Disorders" ) OR ( "Psychiatric Disorder" ) OR ( "Behavior Disorders" ) OR ( "Diagnosis, Psychiatric" ) OR ( "Psychiatric Diagnosis" ) OR ( "Mental Disorders, Severe" ) OR ( "Mental Disorder, Severe" ) OR ( "Severe Mental Disorder" ) OR ( "Severe Mental Disorders" ) ) AND ( ( "telemedicine" ) OR ( "Mobile Health" ) OR ( "Health, Mobile" ) OR ( "mHealth" ) OR ( "Telehealth" ) OR ( "eHealth" ) ) ) AND ( "accept*" ) ) | 1353 |
| SAGE | "Mobile Health" OR "Health, Mobile" OR "mHealth" OR "Telehealth" OR "eHealth") AND (Accept*) AND ("Mental Disorder" OR "Mental Disorders" OR "Psychiatric Illness" OR "Psychiatric Illnesses" OR "Psychiatric Diseases" OR "Psychiatric Disease" OR "Mental Illnesses" OR "Mental Illness" OR "Illness, Mental" OR "Psychiatric Disorder" OR "Psychiatric Disorders" OR "Behavior Disorders" OR "Behavior Disorder" OR "Diagnosis, Psychiatric" OR "Psychiatric Diagnosis" OR "Mental Disorder, Severe" OR "Severe Mental Disorder" OR "Severe Mental Disorders" OR "Telepsychiatry") | 826 |
| Google Scholar | ("Mobile Health" OR "Health, Mobile" OR "mHealth" OR "Telehealth" OR "eHealth") AND (("Mental Disorder "OR "Psychiatric Illness" OR "Psychiatric Illnesses" OR "Psychiatric Diseases" OR "Psychiatric Disease" OR "Mental Illness" OR "Mental Illness" | 370 |
| Scopus | ("Mobile Health" OR "Health, Mobile" OR "mHealth" OR "Telehealth" OR "eHealth") AND (Accept*) AND ("Mental Disorder "OR "Mental Disorders" OR "Psychiatric Illness" OR "Psychiatric Illnesses" OR "Psychiatric Diseases" OR "Psychiatric Disease" OR "Mental Illnesses" OR "Mental Illness" OR "Illness, Mental" OR "Psychiatric Disorder" OR "Psychiatric Disorders" OR "Behavior Disorders" OR "Behavior Disorder" OR "Diagnosis, Psychiatric" OR "Psychiatric Diagnosis" OR "Mental Disorder, Severe" OR "Severe Mental Disorder" OR "Severe Mental Disorders") | 309 |
| Web of science | (("Telemedicine" OR "Mobile Health" OR "Health, Mobile" OR "mHealth" OR "Telehealth" OR "eHealth") AND Accept* AND ("Mental Disorder "OR "Mental Disorders" OR "Psychiatric Illness" OR "Psychiatric Illnesses" OR "Psychiatric Diseases" OR "Psychiatric Disease" OR "Mental Illnesses" OR "Mental Illness" OR "Illness, Mental" OR "Psychiatric Disorder" OR "Psychiatric Disorders" OR "Behavior Disorders" OR "Behavior Disorder" OR "Diagnosis, Psychiatric" OR "Psychiatric Diagnosis" OR "Mental Disorder, Severe" OR "Severe Mental Disorder" OR "Severe Mental Disorders")) | 210 |
| PubMed Medline | ("Mobile Health" OR "Health, Mobile" OR "mHealth" OR "Telehealth" OR "eHealth") AND (Accept*) AND ("Mental Disorder "OR "Mental Disorders" OR "Psychiatric Illness" OR "Psychiatric Illnesses" OR "Psychiatric Diseases" OR "Psychiatric Disease" OR "Mental Illnesses" OR "Mental Illness" OR "Illness, Mental" OR "Psychiatric Disorder" OR "Psychiatric Disorders" OR "Behavior Disorders" OR "Behavior Disorder" OR "Diagnosis, Psychiatric" OR "Psychiatric Diagnosis" OR "Mental Disorder, Severe" OR "Severe Mental Disorder" OR "Severe Mental Disorders") | 203 |
| EBSCO | ("Mobile Health" OR "Health, Mobile" OR "mHealth" OR "Telehealth" OR "eHealth") AND (Accept*) AND ("Mental Disorder "OR "Mental Disorders" OR "Psychiatric Illness" OR "Psychiatric Illnesses" OR "Psychiatric Diseases" OR "Psychiatric Disease" OR "Mental Illnesses" OR "Mental Illness" OR "Illness, Mental" OR "Psychiatric Disorder" OR "Psychiatric Disorders" OR "Behavior Disorders" OR "Behavior Disorder" OR "Diagnosis, Psychiatric" OR "Psychiatric Diagnosis" OR "Mental Disorder, Severe" OR "Severe Mental Disorder" OR "Severe Mental Disorders") | 95 |
